# Passive Smoking Exposure from Partners as a Risk Factor for ER+/PR+ Double Positive Breast Cancer in Never-Smoking Chinese Urban Women: A Hospital-Based Matched Case Control Study

**DOI:** 10.1371/journal.pone.0097498

**Published:** 2014-05-27

**Authors:** Jian-hua Tong, Zhi Li, Jing Shi, He-ming Li, Yan Wang, Ling-yu Fu, Yun-peng Liu

**Affiliations:** Department of Medical Oncology, The First Hospital, China Medical University, Shenyang, China; University Medical Center Utrecht, The Netherlands

## Abstract

**Background:**

The relationship between passive smoking exposure (PSE) and breast cancer risk is of major interest.

**Objective:**

To evaluate the relationship between PSE from partners and breast cancer risk stratified by hormone-receptor (HR) status in Chinese urban women population.

**Design:**

Hospital-based matched case control study.

**Setting:**

Chinese urban breast cancer patients without current or previous active smoking history in China Medical University 1st Hospital, Liaoning Province, China between Jan 2009 and Nov 2009.

**Patients:**

Each breast cancer patient was matched 1∶1 with healthy controls by gender and age (±2 years) from the same hospital.

**Measurements:**

The authors used unconditional logistic regression analyses to estimate odds ratio for women with PSE from partners and breast cancer risk.

**Results:**

312 pairs were included in the study. Women who endured PSE had significantly increased risk of breast cancer (adjusted OR: 1.46; 95% CI: 1.05–2.03; P = 0.027), comparing with unexposed women. Women who exposed to >5 cigarettes/day also had significant increased risk (adjusted OR: 1.99; 95% CI: 1.28–3.10; *P* = 0.002), as were women exposed to passive smoke for 16–25 years (adjusted OR: 1.87 95% CI: 1.22–2.86; *P* = 0.004), and those exposed to > 4 pack-years (adjusted OR: 1.71 95% CI: 1.17–2.50; P = 0.004). Similar trends were significant for estrogen receptor (ER)/progesterone receptor (PR) double positive subgroup(adjusted OR: 1.71; 2.20; 1.99; 1.92, respectively), but not for ER+/PR−, ER−/PR+, or ER−/PR− subgroups.

**Limitations:**

limitations of the hospital-based retrospective study, lack of information on entire lifetime PSE and low statistical power.

**Conclusions:**

Our findings provide further evidence that PSE from partners contributes to increased risk of breast cancer, especially for ER/PR double positive breast cancer, in Chinese urban women.

## Introduction

Breast cancer is the most common neoplasm among women, with more than one million newly diagnosed cases and nearly 600,000 cancer death worldwide annually [1]. Breast cancer is a heterogeneous disease defined by distinct hormone receptors (HR): estrogen receptor (ER) and progesterone receptor (PR) status [2]. HR status is a common and crucial biologic marker in treatment recommendations and prognostic evaluation [3]. Epidemiological risk models show the diverse effects of HR status on breast cancer in evaluating associated risk factors [4], [5]. Although dozens of case-control studies and prospective cohort studies on this topic were done for western populations [6]–[10], similar data are limited for Asians, especially for Chinese. Evaluation of risk factors associated with breast cancer stratified by HR status in China is thus necessary.

Compared with the western countries, breast cancer in China shows more invasive ductal carcinoma with larger tumor size, later stage, lower ER and PR expression and higher HER2 over-expression. This difference was thought to be associated with the different ethnic background and the modifiable lifestyle risk factors between West and East [11]. Recently, investigations of the role of the latter for breast cancer have been spurred by rapid increases in breast cancer rates in China, because researchers consider it due to shifts in risk factor profiles during economic development and westernized life style [12]. On the other hand, several studies show that breast cancer incidence among Asian immigrants to the United States (US) approaches US rates after several generations [13], [14], which implies that potentially modifiable environmental exposure, rather than genetic factors, accounts for differences in breast cancer occurrence.

Unlike most other modifiable lifestyle risk factors, cigarette smoking, including both active smoking and passive smoking exposure (PSE), doesn't actually show consistent association with breast cancer risk in western populations. And the evidence of PSE remains controversial and unclassifiable because of a lack of conclusive mechanistic information [15]. Also, relatively very few studies that compared associations between breast cancer risk factors and HR status have conflicting results [16]–[18]. Additionally, few and controversial studies focused on passive smoking, which were often endured by Chinese urban women from their partners [19]–[24].

Our primary objectives were to determine the relationship between PSE and breast cancer risk, stratified by hormone receptor status in Chinese urban women using data from a hospital-based case-control study.

## Methods

### Ethics Statement

The study's protocol was approved by the hospital human research ethics committee. All the participants provided written informed consent before enrollment.

### Study Population

A hospital-based matched case-control study of breast cancer risk was conducted in the First Hospital of China Medical University from Jan 2009 to Nov 2009. All participants were Chinese women residents, living in the Central Liaoning city cluster, aged 18 years or older.

Case participants were women histologically diagnosed with primary invasive breast cancer based on the 2003 WHO classification of breast cancer, who received surgery in this hospital and were recruited within 6 months from pathologic diagnosis for their interviews. All information on HR status was verified by pathology reports from the Clinical Pathology Diagnosis Center in this hospital, which classified ER and PR status as negative (IHC −), or positive (IHC +, ++ or +++). Controls were women recruited randomly from Medical Examination Centre in this hospital during the same period according to key inclusion criteria, and anyone who was diagnosed as any benign or neoplastic breast disease, or another malignant disease were excluded. Controls were individually matched to cases as 1∶1 by age (up to 2 years older or younger) and sex.

### Data Collection

Study participants were interviewed face-to-face by trained personnel using a structured questionnaire. Each interview usually took 40–50 minutes. Participants were asked about a variety of information including: (1) demographic and basic characteristics, such as living area, education, marital status, height and weight; (2) menstrual, contraceptive and reproductive histories; (3) lifestyle factors, such as alcohol consumption, exercise activity and active smoking histories; (4) medical history, such as family history of cancer; (5) for PSE from partner, we asked these questions: (a) How many people living with you smoke cigarettes and/or pipes? (b) How many cigarettes per day were smoked by your partner with whom you live? (c) How long were you exposed to these smokers?

All data were checked for completeness and authenticity at the end of each interview by a trained researcher. Those who could not recall their smoking exposure histories or refused to report associated information were excluded. Information bias may influence the association between PSE and breast cancer risk, such as women living with different numbers of family smokers; regular or irregular smokers; or women were active smokers also receiving PSE. We defined the definition of passive smoker as women reported living with a partner, who smoked regularly at home for at least one year, as passive smokers. Women who lived with two or more smokers were excluded from the study. Women who reported smoking 20 packs of cigarettes or more over her lifetime were defined as active smokers and also excluded from this study.

### Quantitative variables

Associated PSE variables (PSE, number of cigarettes per day, years of PSE duration and cigarette pack-years) were estimated in separate models. Cigarette pack-years were calculated by multiplying the number of cigarette packs per day to which the subject was exposed by the number of years she was exposed. No PSE were taken as the reference; other levels of number of passive cigarettes per day (0, 1–5, >5), years of PSE duration (0, 1–15, 16–25, >25) and pack-years (0, 0.1–4, >4) were assessed respectively.

Women's routine exercise activity was measured in weekly metabolic equivalent task (MET) hours; MET scores 6, 4.5 and 2.5 were categorized as strenuous sports, moderate activity and walking. MET-hour per week for each intensity activity was expressed as MET score multiplied activity time [25], [26]. Body mass index (BMI, kg/m^2^) at five years before interview date was calculated as weight (kg)/height (m^2^).

### Statistical Methods

Differences in baseline characteristics were assessed by univariate analysis to select potential confounders, including age at interview, educational level (junior high school or less, senior high school or more), age (in years) at menarche (<13, ≥13), parity (0, 1, ≥2), oral contraceptive use (no/yes), family history of cancer (no/yes), exercise activity (MET- hours/week, <9, ≥9), BMI at five years before interview date, alcohol consumption (no/yes). Confounders were reevaluated in multivariate models to further assess associations between PSE and breast cancer risk. These confounders were selected on the basis of previous established risk factors and potential confounders in this study, including age at interview, age at menarche, menopausal status, oral contraceptive use, family history of cancer, alcohol consumption and BMI.

The total samples were then stratified by menopausal status (premenopausal and postmenopausal subgroups). Associations between PSE and breast cancer risk were further assessed in each subgroup. Additionally, we stratified all cases into ER+ or ER− subgroups, and compared PSE-related risks among these 2 subgroups and controls.

All statistical analyses were performed with the SPSS 16.0 statistical package (SPSS Inc., Chicago, IL). In univariate analyses, statistical comparisons were assessed using two-tailed chi-square test for categorical variables, *t*-test for continuous variables and Wald test for trends [27], [28]. Odds ratios (OR) and 95% confidence intervals (CIs) for each level of risk factors were calculated using unconditional logistic regression.

## Results

### Baseline characteristics of study population

The study closed on Nov 30, 2009. Out of the 327 eligible case patients, 319 were interviewed, 8 patients refused to participate in the study (non-response rate: 2.4%). Three hundred thirty-nine eligible controls were recruited, and 329 were interviewed finally (non-response rate: 3.0%). The 7(2.2%) cases and 17 (5.2%) controls who could not remember their smoking exposure histories or who had active smoking histories were excluded from our analyses. Finally, data from 312 eligible patients and 312 matched controls as total samples were analyzed. As only 41 ER−/PR+ patients and 20 ER+/PR− patients were included, making them unsuitable for separate analysis, these two kinds of cases were combined to form a single case group in sub-analyses.

Comparisons of baseline characteristics and exposure factors inpatients and controls are summarized in Table 1 and Table 2. Compared to controls, patients were more likely to have menarche before 13 years of age (19.2% vs 9.9%, *P* = 0.001), to use oral contraceptives (10.6% vs 4.2%, *P* = 0.002), to have first-degree family histories of cancer (23.4% vs 9.3%, *P*<0.001) and lower BMIs (23.4 vs 24.0, *P* = 0.013), but fewer were alcohol drinkers (20.5% vs 36.2%, *P*<0.001). Obvious differences were also seen for PSE. More patients endured PSE at home (59.3% vs 48.1%, *P* = 0.005). However, there were no differences between patients and controls in age at interview, menopausal status, education level, parity, routine exercise activity in terms of MET-hours per week.

**Table 1:** Baseline characteristic of all cases and controls.

Characteristic	Case	Control	P value^a^
	(n = 312)	(n = 312)	
Age at interview (years) (mean ± SD)	48.6±8.66	48.9±8.53	0.713
Education level			0.178
Junior high school or below	78(25.0%)	93(29.8%)	
Senior high school or above	234(75.0%)	219(70.2%)	
Family history of cancer (first-degree relatives)			<0.001
No	73 (23.4%)	29 (9.3%)	
Yes	239(76.6%)	283(90.7%)	
Age at menarche, years			0.001
<13	60 (19.2%)	31 (9.9%)	
≥13	252(80.8%)	281(90.1%)	
Menopausal status			0.626
Premenopausal	180(57.7%)	187(59.9%)	
Postmenopausal	132(42.3%)	125(40.1%)	
Parity			0.175
0	13(4.2%)	24(7.7%)	
1	219(70.2%)	212(67.9%)	
≥2	80(25.6%)	76(24.4%)	
BMI (kg/m^2^) (mean ± SD)	23.4±2.36	24.0±3.49	0.013

a Two-tailed, chi-square test for categorical variables and t-test for continuous variables.

**Table 2:** Exposure factors among Cases and Controls.

Exposure factors	Case	Control	P value^a^
	(n = 312)	(n = 312)	
Oral contraceptive use			0.002
No	279(89.4%)	299(95.8%)	
Yes	33 (10.6%)	13 (4.2%)	
Exercise activity (MET-hours/week)			0.134
<9	213(68.3%)	230(73.7%)	
≥9	99(31.7%)	82(26.3%)	
PSE			0.005
No	127(40.7%)	162(51.9%)	
Yes	185(59.3%)	150(48.1%)	
Alcohol consumption			<0.001
No	248(79.5%)	199(63.8%)	
Yes	64 (20.5%)	113(36.2%)	

Abbreviations: PSE  =  passive smoking exposure. a Two-tailed, chi-square test for categorical variables.

### PSE and breast cancer risk

In our study, continuous data of each exposure factor was categorized as mentioned in methods. Category boundaries were shown in Table 3. To explore associations between PSE and breast cancer risk, the study performed univariate and multivariate analyses. PSE significantly increased breast cancer risk (adjusted OR: 1.46, 95% CI: 1.05–2.03, *P* = 0.027) (Table 4). Compared with controls, women with PSE< 5 cigarettes per day, and those with PSE ≥ 5 cigarettes per day, had 1.21-fold (95% CI: 0.83–1.76), and 1.99-fold (95% CI: 1.28–3.10) increased risks respectively (*P* = 0.010). Duration of PSE was also correlated with breast cancer risk. Compared with controls, women with PSE less than or equal to 15 years, and those with PSE over 16–25 years had 1.60-fold (95% CI: 0.97–2.64) and 1.87-fold (95% CI: 1.22–2.86) greater risk of breast cancer. However, no association was seen in women with >25 years of PSE history (adjusted OR  = 0.92, 95% CI: 0.55–1.53, *P* = 0.743). Cumulative PSE was also significantly associated with breast cancer risk. Compared with controls, adjusted ORs (95% CIs) of women with PSE <4 pack-years, and those with PSE of ≥ 4 pack-years, were 1.12 (0.72–1.76) and 1.71 (1.17–2.50) respectively (*P* = 0.019). Thus, we found evidence of a dose-response trend in breast cancer risk for amount per day, duration and cumulative amount of PSE.

**Table 3:** Category boundaries of all cases and controls.

Factors	Case	Control
	(n = 312)	(n = 312)
Age at menarche, years		
<13	60 (19.2%)	31 (9.9%)
≥13	252(80.8%)	281(90.1%)
Parity		
0	13(4.2%)	24(7.7%)
1	219(70.2%)	212(67.9%)
≥2	80(25.6%)	76(24.4%)
Exercise activity (MET-hours/week)		
<9	213(68.3%)	230(73.7%)
≥9	99(31.7%)	82(26.3%)
Amount of PSE, cigarettes per day		
0	126(40.4%)	161(51.6%)
1–5	98(31.4%)	103(33.0%)
>5	88(28.2%)	48(15.4%)
Duration of PSE, years		
<1	126(40.4%)	161(51.6%)
1–15	54(17.3%)	43(13.8%)
16–25	89(28.5%)	58(18.6%)
>25	43(13.8%)	50(16.0%)
Cumulative amount of PSE, pack-years		
0	126(40.4%)	161(51.6%)
0.1–4	59(18.9%)	69(22.1%)
>4	127(40.7%)	82(26.3%)

Abbreviations: PSE  =  passive smoking exposure

**Table 4:** Association between passive smoking exposure and breast cancer risk.

	Case		Control		OR^a^	95% CI	P value
	(n = 312)		(n = 312)				
	N	%	N	%			
PSE							
No	127	40.7	161	51.6	1.0	Ref^b^	
Yes	185	59.3	151	48.4	1.46	1.05–2.03	0.027
Amount of PSE, cigarettes per day							
0	126	40.4	161	51.6	1.0	Ref^b^	
1–5	98	31.4	103	33.0	1.21	0.83–1.76	0.334
>5	88	28.2	48	15.4	1.99	1.28–3.10	0.002
P_trend_ c							0.010
Duration of PSE, years							
<1	126	40.4	161	51.6	1.0	Ref^b^	
1–15	54	17.3	43	13.8	1.60	0.97–2.64	0.068
16–25	89	28.5	58	18.6	1.87	1.22–2.86	0.004
>25	43	13.8	50	16.0	0.92	0.55–1.53	0.743
P_trend_ c							0.012
Cumulative amount of PSE, pack-years							
0	126	40.4	161	51.6	1.0	Ref^b^	
0.1–4	59	18.9	69	22.1	1.12	0.72–1.76	0.772
>4	127	40.7	82	26.3	1.71	1.17–2.50	0.004
P_trend_ c							0.019

Abbreviations: PSE  =  passive smoking exposure; CI  =  confidence interval; OR  =  odds ratio.

a Adjusted for age at interview, age at menarche, menopausal status, oral contraceptive use, family history of cancer, alcohol consumption and BMI.

b Reference category.

c Wald test for trend in case-control analyses.

The association between PSE and breast cancer risk was assessed by menopausal status and further compared in Table S1. According to diaries of PSE exposure and cigarette pack-years, ORs in the premenopausal subgroup were consistent with their counterparts in the total samples.

### PSE and breast cancer risk by ER/PR status

We further explored the relationship between PSE and breast cancer risk by HR status. This trend was only observed in patients with ER+/PR+ group (Table 5). Relative to controls, women with PSE< 5 cigarettes per day, and those with PSE ≥ 5 cigarettes per day, had increased breast cancer risk with ORs of 1.42 (95% CI: 0.91–2.22) and 2.20 (95% CI: 1.32–3.65), respectively, in the ER+/PR+ group (*P* = 0.009). Relative to controls, adjusted ORs (95% CIs) were 1.91 (1.08–3.38), 1.99 (1.21–3.27), and 1.17 (0.65–2.09) in women having PSE for < 15 years, 16–25 years, and > 25 years, respectively (*P* = 0.021). Relative to controls, a significant dose-response relationship between cumulative amount of PSE and the risk of HR double positive breast cancer was also shown in women with PSE < 4 pack-years, and those with PSE of ≥ 4 pack-years, whose adjusted ORs (95% CIs) were 1.37 (0.82–2.29) and 1.92 (1.23–3.00) (*P* = 0.015). On the contrary, there were null associations on all passive smoking factors in ER+/PR−, ER−/PR+, and ER−/PR− patients (*P*>0.10), but the statistical power was limited by the small sample sizes. Women with PSE ≥25 years had lower risk of breast cancer than did the control group (adjusted OR  =  0.28, 95% CI: 0.09–0.86, *P* = 0.035). To sum up, a weak but significant positive relationship between PSE and breast cancer risk was seen for the ER+/PR+ subtype; however, risks to other subtypes are difficult to determine due to limited statistical power.

**Table 5:** Relationship between passive smoking exposure and breast cancer risk by ER/PR status.

		ER+/PR+		ER+/PR− or ER−/PR+		ER−/PR−	
	No.	No.	OR^a^	No.	OR^a^	No.	OR^a^
	Controls	Cases	(95%CI)	Cases	(95%CI)	Cases	(95%CI)
Passive smoking exposure							
No	161	66	1.0^b^	28	1.0^b^	33	1.0^b^
Yes	151	115	1.71(1.16–2.53)	33	1.07 (0.59–1.94)	37	1.03 (0.59–1.79)
Amount of PSE, cigarettes per day							
0	161	66	1.0^b^	27	1.0^b^	33	1.0^b^
1–5	103	62	1.42(0.91–2.22)	16	1.00(0.49–2.02)	20	0.90(0.48–1.69)
>5	48	53	2.20 (1.32–3.65)	18	1.42(0.67–3.02)	17	1.26 (0.61–2.60)
P_trend_ c			0.009		0.615		0.689
Duration of PSE, years							
<1	161	66	1.0^b^	27	1.0^b^	33	1.0^b^
1–15	43	37	1.91 (1.08–3.38)	6	0.91 (0.32–2.62)	11	1.42(0.61–3.2229)
16–25	58	51	1.99(1.21–3.27)	16	1.65(0.78–3.51)	22	1.60(0.82–3.119)
>25	50	27	1.17 (0.65–2.09)	12	0.88 (0.38–2.05)	4	0.28(0.09–0.86)
P_trend_ c			0.021		0.512		0.035
Cumulative amount of PSE, pack-years							
0	161	66	1.0^b^	27	1.0^b^	33	1.0^b^
0.1–4	69	41	1.37(0.82–2.29)	7	0.67 (0.26–1.71)	11	0.78(0.36–1.70)
>4	82	74	1.92(1.23–3.00)	27	1.47 (0.77–2.82)	26	1.19 (0.64–2.19)
P_trend_ c			0.015		0.24		0.608

Abbreviations: PSE  =  passive smoking exposure; CI  =  confidence interval; OR  =  odds ratio.

a Adjusted for age at interview, age at menarche, menopausal status, oral contraceptive use, family history of cancer, alcohol consumption and BMI.

b Reference category.

c Wald test for trend in case-control analyses.

To improve the statistical power, we compared PSE-related risks among the ER+ subgroup, the ER−subgroup and controls. In this analysis, PSE remained a significant risk factor when comparing the ER+ subgroup with controls, but was not a significant risk factor when we compared risks in ER– subgroup with its control group, or the ER+ subgroup with the ER− subgroup (Table S2).

## Discussion

Recent studies suggest that PSE is no safer than active smoking [29]–[32], which implies that worldwide estimation of PSE and its effects are important. Although few Chinese urban women are active smokers [24], [33], at least half of them endure PSE [34], [35], primarily from their partners [19]–[22]. Furthermore, whereas alcohol consumption is thought to be the greatest confounding factor in evaluating smoking effects among western populations, Chinese urban women consume relatively little alcohol. Thus Chinese urban women are a particularly appropriate population in whom to estimate relationships between PSE from partners and breast cancer risk. In this study, various measures of PSE were significantly related to breast cancer risk in a dose-response manner,which supported the hypothesis that PSE is associated with increased breast cancer risk among them.

These data are consistent with many previous epidemiological studies [29], [30], [36]–[40], which indicate, but do not entirely prove, a causal relationship. On the other hand, many reports had null results on this topic, including some well-designed, large, prospective cohort studies [15], [16], [22], [41]–[46]. Additionally, the recent meta-analysis, which based on some perspective studies, also got the null associations between PSE and breast cancer risk [47], [48]. However, some problems were still not resolved: 1) breast cancer was considered to have great heterogeneity, different cancers had unique and different sensitivity to ER-related pathway. Thus it is hard to explain with unified “anti-estrogen effect” [3]. 2) The study populations had complex PSE, including PSE in the childhood, the adolescence, the manhood (before and after menopause, and perimenopause). Estrogen should have different physiological effects during different periods [7]. 3) The specialty of breast cancer was different between West and East, and there were more invasive ductal carcinoma patients with larger tumor size, later stage, lower HR expression and higher HER2 over-expression in Chinese population [11]. 4) The technique of meta-analysis was imperfect: i. it was controversy to select fixed/random effect model with I-square and Q-test. Some researchers believed that the clinical sense was more important; ii. Breast cancer occurrences were rare events in the prospective studies, but no adjustments were mentioned in the meta-analysis. iii. Both studies were with some unexplained and extreme heterogeneity.

All the above hinted that there might not existed an identical effect of PSE on breast cancer risk. And we held an opinion that the relationship might depend on ethnicity, breast cancer heterogeneity, and other underlying factors. Therefore future researches should identify high-risk groups among the entire PSE population [49]; and authenticate pathologic types among the entire population of patients with breast cancer. In the study, Chinese urban women were shown to be a high-risk group, which offered a clue to the etiology. It was consistent with a recent meta-analysis, which included some retrospective studies in Chinese population [23]. But the conclusion from this meta-analysis should be considered very carefully, based on the following reasons: 1) Some original studies might be with low-quality, and the details of quality assessment were not provided by the authors. 2) As the authors mentioned in the discussion part, there was an “unavoidable publication bias” in the meta analysis. 3) The study had some unexplained and extreme heterogeneity according to the text description and the forest plots in the results part, either. So we supposed that it was still valuable to perform single case-control study on this topic and hoped that some high-quality meta-analysis with individual participant data, or at least stratified by PSE from partners, could provide further evidence in the future.

Moreover, the current data suggest that HR+ breast cancer is particularly associated with PSE, which was consistent with some previous studies of western patients [16], [17], [50], [51].This point is supported by recent reports that sidestream smoke contains higher concentrations of nicotine than does mainstream smoke; and that nicotine has a strong synergistic effect on the ER pathway, shown in both inflammatory disease and cancer [52]–[54].However, few investigations have focused on this topic in a Chinese population. Here, our study implies an important association between PSE and HR+ breast cancer, which is consistent with the lower HR+ breast cancer incidence and lower rate of cigarette smoking in Chinese urban women, compared with western populations. The topic clearly warrants further investigation.

In this study, a weak dose-response trend was seen only in the population who endured ≤25 years of PSE history. Notably those with >25 years of PSE history, the association was null or even slightly inverse (Tables 2 & 3). This appeared to reflect the expert panel's opinion that a possible causal relationship between PSE and breast cancer existed mostly among younger, primarily premenopausal women [49], [55].We conducted a sub-group analysis stratified by menopausal status, and obtained consistent results especially between the premenopausal subgroup and total samples (Table S1), which further supported our opinion in this matter. It is also noticed that participant numbers were small and confidence intervals were wide in the current study, especially in sub-group analysis, which implied underpowered statistics, and the need for confirmation in a larger, well-designed prospective cohort study in the future.

The strength of the study was the strict criteria of recruitment and grouping: Participants were all city dwellers, who were presumed to have similar lifestyles. Patients and controls were 1: 1 matched by age (+/− 2 years), which granted intergroup balance as much as possible. We also evaluated PSE quantitatively, which was uncommon compared with the uncertainty of PSE intensity in the previous studies [22], [40], [46]. To assure the precision of the current study, we only evaluated those who lived with no or only one regular active smoker (their husbands), and excluded subjects who could not provide sufficient data on their exposure to PSE.

One limitation of the study was its retrospective nature, leading to possible memory bias, especially for recalling partners' behavior among those who were newly diagnosed with cancer. They were more conscious of their disease, and, although they could be more likely to obtain information on their family history and cigarette exposure, were also more prone to exaggerate points that they thought would lead to their disease. This situation may overstate the risk. Additionally, given that each participant reported her husband's smoking behavior, the report of passive smoking, especially as to dose, is a proxy exposure, which might decrease the accuracy. Third, this study was a hospital-based, rather than population-based study, which potentially implies a selection bias. However, recent studies, using the data from this hospital, show that hospital controls are comparable with population controls for most demographic characteristics and lifestyle factors measured [56]. Fourth, we did not collect subjects' entire lifetime history of PSE, such as in workplaces or in childhood, which might contaminate the control group and underestimate the risk. Fifth, although PSE was significantly associated with HR+ tumors (especially ER+/PR+, the dominant subtype), the statistical power limited us to get affirmative conclusions for other hormone receptor subtypes.

## Conclusion

To clarify the association between PSE from partners and breast cancer risk in a Chinese urban female population, we performed a case-control study that included 312 breast cancer patients and 312 controls, matched at a 1∶1 ratio by age and residence. We found that PSE was associated with increased risk of breast cancer, with a weak dose-response relationship between PSE and breast cancer, especially in ER+/PR+ cancers. The association between PSE from partners and ER/PR-related breast cancer warrants further study.

## Supporting Information

Table S1 **Association between passive smoking exposure and breast cancer risk.**
(DOC)

Table S2 **Relationship between passive smoking exposure and breast cancer risk by ER/PR status.**
(DOC)
