# Supplementary material for: Passive Smoking Exposure from Partners as a Risk Factor for ER+/PR+ Double Positive Breast Cancer in Never-Smoking Chinese Urban Women: A Hospital-Based Matched Case Control Study
Source: PLoS One. 2014 May 27;9(5):e97498. doi: 10.1371/journal.pone.0097498 (PMC4035255; doi:10.1371/journal.pone.0097498)
Supplement: Table S1 — Association between passive smoking exposure and breast cancer risk. (DOC) [file pone.0097498.s001.doc]

**Supplementary Table 1. Association between passive smoking exposure and breast cancer risk**

Abbreviations: CI=confidence interval; OR=odds ratio.

|  | **Total samples** | |  | **Premenopausal** | |  | **Postmenopausal** | |
| --- | --- | --- | --- | --- | --- | --- | --- | --- |
|  | **OR^a^** | **P value** |  | **OR^a^** | **P value** |  | **OR^a^** | **P value** |
| Passive smoking exposure  No  Yes | 1.0  1.46 | 0.027 |  | 1.0^b^  1.43 | 0.112 |  | 1.0^b^  1.42 | 0.189 |
| Average number of passive  cigarettes per day  0  1-5  >5  P _trend_^c^ | 1.0  1.21  1.99 | 0.334  0.002  0.010 |  | 1.0^b^  1.09  2.44 | 0.746  0.004  0.013 |  | 1.0^b^  1.36  1.50 | 0.321  0.238  0.408 |
| Number of passive smoking  exposure years  <1  1-15  16-25  >25  P _trend_^c^ | 1.0  1.60  1.87  0.92 | 0.068  0.004  0.743  0.012 |  | 1.0^b^  1.42  1.52  1.24 | 0.257  0.124  0.635  0.423 |  | 1.0^b^  2.51  2.30  0.87 | 0.073  0.030  0.676  0.039 |
| Cigaretee pack-years  0  0.1-4  >4  P _trend_^c^ | 1.0  1.12  1.71 | 0.772  0.004  0.019 |  | 1.0^b^  1.00  2.06 | 0.992  0.009  0.020 |  | 1.0^b^  1.69  1.34 | 0.218  0.301  0.368 |

**a** Adjusted for age at interview, age at menarche, oral contraceptive use, family history of cancer, alcohol consumption and BMI.

**b** Reference category.

**c** Wald statistic test for trend from case-control analyses.
