# Supplementary material for: Passive Smoking Exposure from Partners as a Risk Factor for ER+/PR+ Double Positive Breast Cancer in Never-Smoking Chinese Urban Women: A Hospital-Based Matched Case Control Study
Source: PLoS One. 2014 May 27;9(5):e97498. doi: 10.1371/journal.pone.0097498 (PMC4035255; doi:10.1371/journal.pone.0097498)
Supplement: Table S2 — Relationship between passive smoking exposure and breast cancer risk by ER/PR status. (DOC) [file pone.0097498.s002.doc]

Supplementary Table 2. Relationship between passive smoking exposure and breast cancer risk by ER/PR status

|  | **ER+ vs. ER-** | | |  | **ER+ vs. Controls** | | | |  | | **ER- vs. Controls** | | | |  |
| --- | --- | --- | --- | --- | --- | --- | --- | --- | --- | --- | --- | --- | --- | --- | --- |
|  | **No.**  **ER+** | **No.**  **ER-** | **OR^a^**  **(95%CI)** |  | **No.**  **ER+** | **No.**  **Cons** | | **OR^a^**  **(95%CI)** | |  | | **No.**  **ER-** | **No.**  **Cons** | **OR^a^**  **(95%CI)** | |
| Passive smoking exposure  No  Yes | 75  126 | 52  59 | 1.0^b^  1.54(0.95-2.50) |  | 75  126 | 162  150 | 1.0^b^  1.65(1.13-2.41) | | |  | | 33  37 | 162  150 | 1.0^b^  1.02(0.64-1.63) | |
| Average number of passive cigarettes per day  0  1-5  >5  P _trend_^c^ | 75  67  59 | 51  31  29 | 1.0^b^  1.50(0.86-2.64)  1.46(0.81-2.63)  0.277 |  | 75  67  59 | 161  103  48 | 1.0^b^  1.38(0.90-2.12)  2.12(1.29-3.47)  0.011 | | |  | | 33  20  17 | 161  103  48 | 1.0^b^  0.93(0.54-1.60)  1.30(0.70-2.40)  0.594 | |
| Number of passive smoking  exposure years  <1  1-15  16-25  >25  P _trend_^c^ | 75  38  58  30 | 51  16  31  13 | 1.0^b^  1.66(0.83-3.35)  1.30(0.73-2.31)  1.73(0.80-3.75)  0.363 |  | 75  38  58  30 | 161  43  58  50 | 1.0^b^  1.82(1.04-3.17)  1.99(1.23-3.20)  1.09(0.61-1.92)  0.018 | | |  | | 33  11  22  4 | 161  43  58  50 | 1.0^b^  1.25(0.61-2.56)  1.50(0.83-2.70)  0.54(0.25-1.16)  0.126 | |
| Cigaretee pack-years  0  0.1 -4  >4  P _trend_^c^ | 75  42  84 | 51  17  43 | 1.0^b^  1.71(0.86-3.39)  1.39(0.82-2.35)  0.237 |  | 75  42  84 | 161  69  82 | | 1.0^b^  1.30(0.79-2.15)  1.87(1.22-2.87)  0.016 | |  | | 33  11  26 | 161  69  82 | 1.0^b^  0.81(0.42-1.58)  1.23(0.73-2.08)  0.496 | |

Abbreviations: CI=confidence interval; OR=odds ratio. Cons=Controls

**a** Adjusted for age at interview, age at menarche, menopausal status, oral contraceptive use, family history of cancer, alcohol consumption and BMI.

**b** Reference category.

**c** Wald statistic test for trend from case-control analyses.
